# Effect of Implicit Learning Methods With the External Focus of Attention on Bowling Skills in Children With Autism Spectrum Disorder: A Randomized Control Trial Study

**DOI:** 10.1002/brb3.70139

**Published:** 2024-12-06

**Authors:** Mina Khodayari, Rasoul Yaali, Farhad Ghadiri

**Affiliations:** ^1^ Faculty of Physical Education and Sport Sciences Kharazmi University of Tehran Tehran Iran

**Keywords:** analogical learning, attention, errorless learning

## Abstract

**Purpose:**

The aim of the current study was to compare implicit learning methods with an emphasis on the external focus of attention on bowling skill in autistic children.

**Method:**

Twenty children with autism spectrum disorder (ASD) were selected. After the participants were randomly divided into two groups, the pretest was performed, evaluating the participants both quantitatively (score of bowling pins falling) and qualitatively (TGMD3 subscale test of underhand ball throwing). Group A was trained using the errorless learning method with the external focus of attention, while Group B was trained using the analogical learning method with the external focus of attention.

**Findings:**

The results showed that analogical learning with the external focus of attention has significant effects (*p* ≤ 0.05) on both bowling and underhand ball‐throwing skills in autistic children. Errorless learning with external focus of attention, on the other hand, had a significant effect (*p* ≤ 0.05) on the bowling skill.

**Conclusion:**

The results of the research showed that analogical learning with an external focus of attention can be effective in developing both bowling skill and underhand ball‐throwing skill in children with ASD; however, errorless learning with an external focus of attention was useful in developing bowling skill and failed to show a significant effect on enhancing the underhand ball‐throwing skill in children diagnosed with ASD.

**Trial Registration:**

IRCT20220920056007N1.

## Graphical abstract

Analogical learning with an external focus of attention can be effective in motor skill learning in children with ASD. This approach is beneficial when combined with errorless training with an external focus of attention.

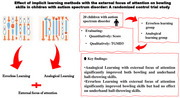

## 1 Introduction

Currently, autism spectrum disorder (ASD) is one of the most basic and important issues at the global level and in the field of mental health. ASD is a developmental disorder that affects a person's ability to communicate, understand language, and interact with others (Dunlap and Bunton‐Pierce 1999). Signs and symptoms of ASD include not being able to understand and process information well, having weak muscle tone, having trouble with motor development, not being able to copy or model movements, having neurocognitive disorders, having trouble with eye and hand movements, not being flexible, attention disorder, and repetitive and restrictive behavior patterns (Bhat, Landa, and Galloway 2011; Greenaway and Davis 2013; Kern et al. 2011; Lolk 2013; Thelen 2000). Attention is one of the most important factors in learning (Schmidt and Wrisberg 2008) and is the cognitive function of focusing on specific targets in the surroundings while ignoring other omitted stimuli (Wolfe 1998). The negative effects of poor motor learning show how important it is to come up with effective ways to improve motor learning and motor performance in people with ASD (Wulf 2013). The modification of one's focus of attention is a potential strategy that has drawn considerable interest. Instructions that get the learner to pay attention to the effects of their movements on the environment, like “pay attention to how your movements affect the world around you,” have been found to work better than those that tell the learner to focus on their movements, like “pay attention to how your fingers move” (Wulf 2013). A review of previous research shows that most children with developmental disorders, including mental retardation and ASD, show deficits in attention (Burack et al. 2001; Kanner 1943; Mottron et al. 2006; Mundy and Neal 2000; Ornitz 1988). The existence of unusual and abnormal responses to environmental stimuli in children with ASD has led to extensive research in the field of attention in people with ASD. Moreover, the analysis of these responses concerning movement skills has been explored to some extent (Tse 2019). Chiviacowsky, Wulf, and Ávila (2013) found that children with intellectual disabilities (IQ = 51–69) learn to use their bodies better when they focus on something outside of themselves. Thus, throwing, kicking, and other goal‐oriented ball games may be a potential way to improve coordination because of the immediate multisensory feedback and external focus of attention that come with these skills (Colebourn, Golub‐Victor, and Paez 2017). Capio et al. (2013) looked at how children with intellectual disabilities use their overhand throwing skills and recommended interventions that limit the environment to reduce the number of output errors.

Motor learning is a relatively stable change in motor behavior achieved with practice and experience (Schmidt and Wrisberg 2008). Motor learning is the acquisition of movement abilities (Maxwell and Masters 2008). Learning is one of the complex cognitive processes that is divided into two main categories, which we know as explicit and implicit learning. Implicit learning is the process of acquiring knowledge without conscious awareness of what is being learned or, at times, even a desire to learn (Perrig 1996; Thorndike and Rock 1934). On the other hand, explicit learning involves complete or partial cognitive awareness of the material being learned and is distinguished by the use of hypothesis testing techniques (Raab et al. 2009). Masters and Maxwell (2004) argued that implicit learning is when a person passively gathers information about a skill that is processed at an unconscious level and cannot be put into words. Explicit learning refers to the process in which a learner has a distinct understanding of the motor knowledge they are acquiring. Conversely, implicit learning involves the acquisition of information without a conscious awareness of the skills learned (Masters 1992). Implicit learning, similarly, would block hypothesis testing in working memory (Masters 1992). The process of hypothesis‐testing behavior involves the creation of movement strategies and their evaluation, using outcome feedback, within the working memory (Baddeley and Hitch 1974). In this capacity, working memory functions as a brief store and manager of explicitly available information, enabling tracking and modification of movements as they occur (Masters and Maxwell 2004). Magill (1998) categorized some of the prevalent types of implicit learning as follows: skill learning while performing dual tasks at once, learning without receiving feedback on the outcome, discovery learning, and errorless learning. Masters (1992) presented analogy learning as a method for lowering explicit information and enhancing implicit motor skill acquisition. These teaching methods have been used in various research studies. Some previous research shows the superiority of implicit learning over explicit learning in children with ASD (Izadi‐Najafabadi et al. 2013; Nemeth et al. 2010; Travers et al. 2010).

Analogy learning is one mode of implicit learning (Liao and Masters 2001; Masters 2000; Poolton, Masters, and Maxwell 2006). Analogy aids in the learning of a new concept by linking it to an essentially equivalent concept (Gentner 1983; Schustack and Anderson 1979). Studies show that analogy learning during motor skill acquisition reduces reliance on information‐cognitive processes (Poolton, Masters, and Maxwell 2006). Learning by analogy necessitates the conversion of knowledge related to a known (but independent) idea into a concept that must be learned (Gentner 1983). Task‐related knowledge is taught through analogy training in the context of motor learning. However, it frequently generates a strong mental visual image that is appealing to the learner (Chatzopoulos et al. 2022). Errorless learning is a method that prevents children from making mistakes and gradually moves stimulus control from manufactured responses to naturally occurring stimuli (Craig 2021). By employing early and immediate prompts that minimize errors during instructions aimed at skill development, errorless learning allows the child to get access to reinforcers (Oppenheimer, Saunders, and Spradlin 1993). Over time, a mechanism is designed to gradually fade out prompts, allowing a child to reply independently (Craig 2021). An external focus of attention can be used to influence implicit motor learning (van Abswoude et al. 2021). Training that shifts the performer's focus beyond his or her own body movements to the consequences that their actions have on their surroundings (external focus) will improve learning (Wulf et al. 1998).

Considering that children with ASD have repetitive movements and can hardly change their behavior from one activity to another (Kern et al. 2013), and based on the benefits and advantages of implicit learning over explicit learning as well as the results of past research (Andy, Wong, and Masters 2017; Kal et al. 2018; Masters, Poolton, and Maxwell 2008; Maxwell et al. 2001; Poolton et al. 2006; Singer, Lidor, and Cauraugh 1993; Totsika and Wulf 2003; Wulf, McNevin, and Shea 2001), this educational method seems like it can lead to enhanced development in children with ASD (Foti et al. 2015; Halsband and Lange 2006; Mostofsky et al. 2000; Rogers et al. 2013). Also, considering the benefits of an external focus of attention to reduce attention and cognitive load in the nervous system and executive functions, the use of external attention can also yield significant effects. Now this question is raised: if implicit learning is used in combination with the external focus of attention on individuals, will the benefits of both approaches synergize and yield greater outcomes? On the other hand, since there have been different suggestions for implicit learning (Lam, Maxwell, and Masters 2009; Liao and Masters 2001; Masters 1992; Maxwell et al. 2001; van Abswoude et al. 2021), which method, when combined with an external focus of attention, helps these individuals learn better? Considering that people with ASD have problems in terms of attention, when we turn attention to the outside (external attention) to teach skills, it creates procedural knowledge that makes the person faster compared to when attention is directed internally (Shea and Wulf 1999; Wulf et al. 1998; Wulf, Shea, and Park 2001). Fundamental movement skills (FMSs) are considered the fundamental components of movement and serve as the basis for many of the specific movement skills required for effective participation in sports and physical activities (Gallahue and Ozmun 2006). Proficiency in FMS is not typically acquired organically; it requires deliberate learning, practice, and development (Gagen and Getchell 2006) FMSs are sometimes classified as locomotor skills, such as running, jumping, and hopping, and object‐control skills, such as catching, throwing, and kicking (Heywood and Getchell 2009). The literature on motor learning indicates that the majority of children, regardless of gender, have the developmental ability to achieve mastery of all FMS by Grade 4 (about 10 years old). This can be achieved by providing activities and equipment that are suitable for their developmental stage, using appropriate visual demonstrations of skills, providing instruction and feedback, offering a range of relevant, enjoyable, and challenging practice activities, and creating a positive learning environment (Gallahue and Ozmun 2006).

Previous studies have demonstrated that teaching children with ASD through games leads to procedural learning. Therefore, the aim of the present study was to compare errorless learning and analogical learning methods with an emphasis on the external focus of attention on the retention and transfer of bowling skills in children with ASD. In this study, we hypothesized that errorless learning by the external focus of attention would have a significant effect on the retention and transfer of bowling skills in children with ASD.

## 2 Methods

### 2.1 Design of the Study

Before data collection, the Iranian Registry of Clinical Trials (IRCT) procedure was approved by the research ethics committee (IR.SSRC.REC.1401.07) and registered (IRCTID: IRCT20220920056007N1). An informed permission form was signed by the parents of every study participant. The training sessions lasted for 6 weeks, and the participants practiced in the rehabilitation center. The recruitment start date was January 28, 2023, and the end date was March 21, 2023.

### 2.2 Participants

The samples of this study included 20 children with ASD aged 5–12 years (M ± SD: Group A: 2.646 ± 8.50; Group B: 2.217 ± 8.75). The inclusion criteria for this study were as follows: absence of vision and hearing impairments, no pathological conditions affecting the upper and lower limbs, and the requirement of being right‐handed. Conversely, the exclusion criteria consist of the lack of a signed consent form, inability to complete the pretest, and unwillingness to participate in the exercises. A research team comprised of experienced child psychiatrists and licensed clinical psychologists confirmed the clinical diagnoses of ASD in accordance with DSM‐5 criteria, using all available clinical information. To verify each diagnosis, we assessed the severity of autistic symptoms using the Autism Diagnostic Interview Revised (ADI‐R) and the Autism Diagnostic Observation Schedule‐2 (ADOS‐2), both administered by interviewers with a proven track record in scientific research.

The participants were randomly divided into two groups through purposeful randomization so that the average age and pretest scores of the two groups did not have a significant difference.

### 2.3 Protocols

In this research, 10 bowling pins with a height of 25 cm and a width of 8 cm were used (Abdollahipour et al. 2017). Bowling pins are designed with cartoon characters (Carter et al. 2014; Chaminade et al. 2015; El‐Seoud, Halabi, and Geroimenko 2018) to draw attention outward. Also, the toy police car, star, frog leaf, fish, and sea were used for training. A record‐keeping checklist was used for pretest and post‐test. Two checklists were used: (1) a checklist for quality assessment (TGMD3) (Webster and Ulrich 2017), and (2) a checklist for quantitative assessment (score of bowling pins falling) (Abdollahipour et al. 2017). The Edinburgh Handedness Inventory was also used to diagnose the dominant hand, as were the Cattell Intelligence Scales 1 and 2 (Table 1) and the Ulrich Gross Motor Development Test, 3rd Edition (TGMD3).

**TABLE 1:** Demographic description of participants.

	Errorless learning group	Analogical group
	mean ± SD	mean ± SD
**Age**	2.646 ± 8.50	2.217 ± 8.75
Cattell intelligence test	121.75 ± 6.238	121.25 ± 6.801

First, a pretest was administered to the participants, who were divided into two groups: Group A (*n* = 10; age: mean ± SD = 2.646 ± 8.50 years; Cattell intelligence test: mean ± SD = 121.75 ± 6.238; history of visiting the center: mean ± SD = 4.0 ± 0.816 years) and Group B (*n* = 10; age: mean ± SD = 2.217 ± 8.75 years; Cattell intelligence test: mean ± SD = 121.25 ± 6.801; history of visiting the center: mean ± SD = 4.0 ± 0.816 years). In this study, training trials were designed in parallel groups.

Group A was trained using the errorless learning method. Also, bowling pins with cartoon characters that were familiar to the participants were used in this method to direct attention to the outside (Table 2). In this method, the participant's distance from the bowling pins was 30 cm, and 10 cm was added to the distance for every two sessions. Group B was trained using the analogical learning method (Table 3). Similar to Group A, for this group, bowling pins with cartoon characters were used as an external focus of attention. In this method, analogy was used to teach skill components: In the initial two sessions, a toy police car was utilized; subsequent sessions involved rescuing fish from cats, stepping on a frog's leaf, and maintaining hand movement by hitting a star after a throw. It is noted that in the transfer test, the participants were given a lighter ball (Van den Tillaar and Marques 2013), and all exercises were performed by the occupational therapist of the rehabilitation center.

**TABLE 2:** Interventions for errorless learning group.

Sessions	Interventions				
Group A	Distance of bowling's pins	Exercises	Repetition exercise trials	External focus of attention	Pictures
1–2	30 cm	Tools: Toy bowling ball for throwing 10 toy bowling pins The occupational therapist holds her hand at a distance of 30 cm parallel to the ground and tells the participant that he should throw the bowling ball from under my hand toward the Red Hat (bowling pins designed with the cartoon character of the Red Hat) so that the ball hits them and falls down. The participant's distance from the first bowling pins was 30 cm, and 10 cm was added to the distance for each session.	20	Toy bowling pins are designed with Red Hat cartoon characters to draw attention outward (external attention). The red pins were 25 cm in height and 8 cm wide at their widest point. They were arranged with 20 cm between each neighboring pin in an equal triangular arrangement.	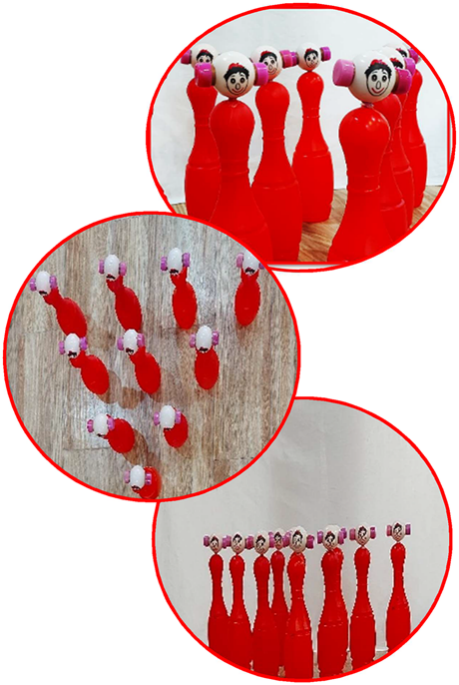
3–4	40 cm		20		
5–6	50 cm		20		
7–8	60 cm		20		
9–10	70 cm		20		
11–12	80 cm		20		
13–14	90 cm		20		
15–16	100 cm		20		

*Note*: All bowling pins were the same color. Participants were previously trained in the concepts of left, right, up, and down in the rehabilitation center. If 80% of the bowling pins were knocked down (eight bowling pins or higher), the next trials were made from the same distance to the bowling. If 80% less were knocked down to the ground, first we reduced the distance to the bowling by 10 cm to reach 80%, and when it reached 80%, we increased the distance by 10 cm again. Cartoon characters, which were familiar to children, were used for bowling pins. The Red Hat cartoon was previously shown in the rehabilitation center for children.

**TABLE 3:** Interventions for analogical learning group.

Session	Intervention				
Group B	Exercises	Repetition exercise trials	External focus of attention	Pictures	
1–2	Distance from bowling: 2 m In this session, the distance from the bowling pins is built in the form of a road. The occupational therapist gave the police car to the participants and told them that the Red Hats (cartoon characters on the bowling pins) were in danger. Let the police rescue them.	20	Toy bowling pins are designed with Red Hat cartoon characters to draw attention outward (external attention).	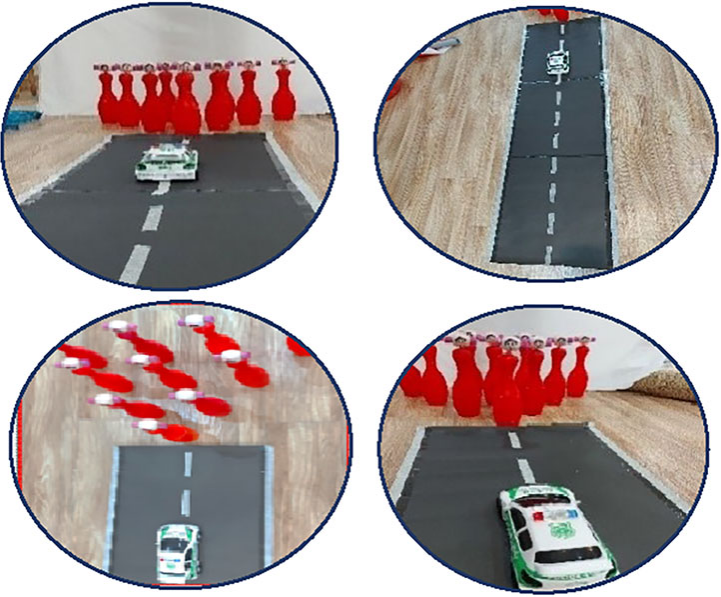	
3–4		20			
5–6 7–8	Distance from bowling: 2 m From this session on, instead of the road, we will replace it with the sea, where a number of fish are located near the bowling pins at a distance of about 1 m. We also give toy bowling balls instead of toy cars. Bowling pins in this session have cat labels (instead of Red Hat cartoon characters). The occupational therapist tells the participants that the cats want to eat fish, and they have to save the fish. To save the fish, they had to hit the cats with this ball so that they were knocked down and could not eat the fish.	20 20	From this session on, the bowling pins were designed with cats instead of a Red Hat, and the participants' attention was drawn to them. The red and blue pins were 25 cm in height and 8 cm wide at their widest point. They were arranged with 20 cm separating neighboring pins in an equal triangular arrangement.	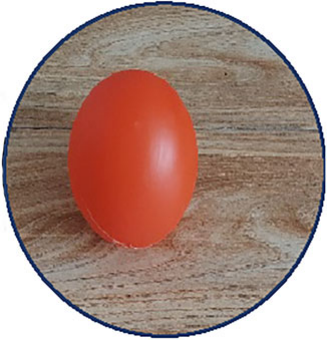	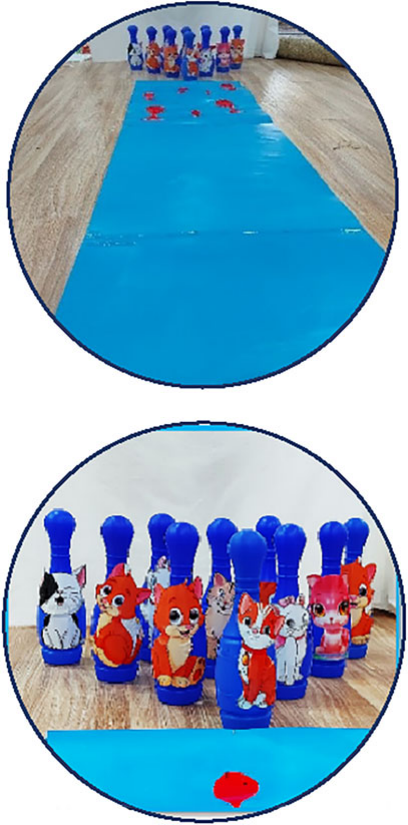
9–10	Distance from bowling: 2 m The frog leaf is placed on the blue cardboard, which we consider the sea, and the occupational therapist asks the participants to place the foot opposite the throwing hand (left foot if they are right‐handed and right foot if they are left‐handed) on the leaf before throwing the ball. Give and then throw the ball at the cats to save the fish.	20		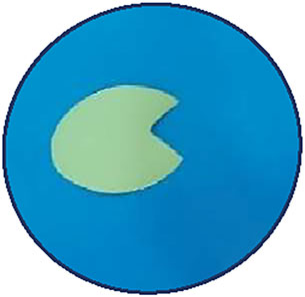	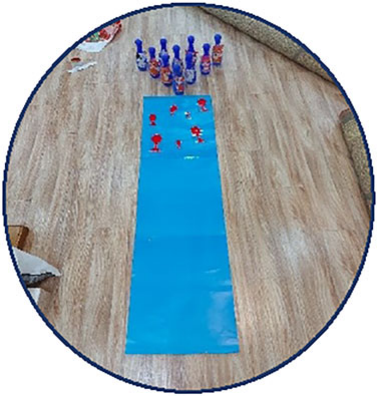
11–12	Distance from bowling: 2 m To continue the movement of the hand, we hold up the wooden stick that is the hanging star and ask the participants to hit the star after throwing the ball.	20		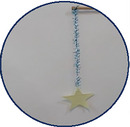	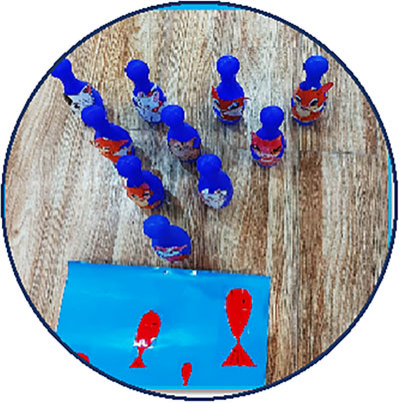
13–14 15–16	Distance from bowling: 2 m Before throwing the ball, put the opposite foot of the throwing hand on the leaf. Throw the ball at the cats to save the fish. To continue the movement of the participant's hand, we pick up the stick with the hanging star and ask the participants to touch the star after throwing it.	20 20		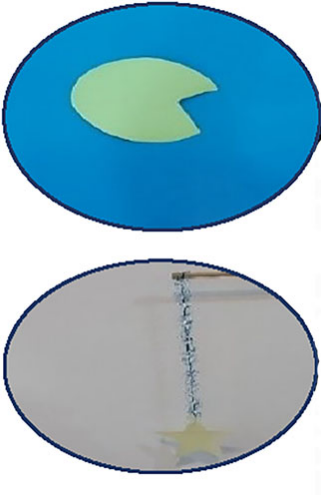	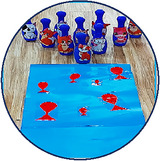

*Note*: All bowling pins were of the same color (red for characters and blue for cats). Subjects were previously trained in the concepts of left, right, up, and down in the rehabilitation center. In the fourth intervention session, subjects were given a toy police car instead of a ball, and from the fifth session on, they were given a ball. Cartoon characters were used for bowling pins, which were familiar to children. The Red Hat cartoon was previously shown in the rehabilitation center for children.

**FIGURE 1:**
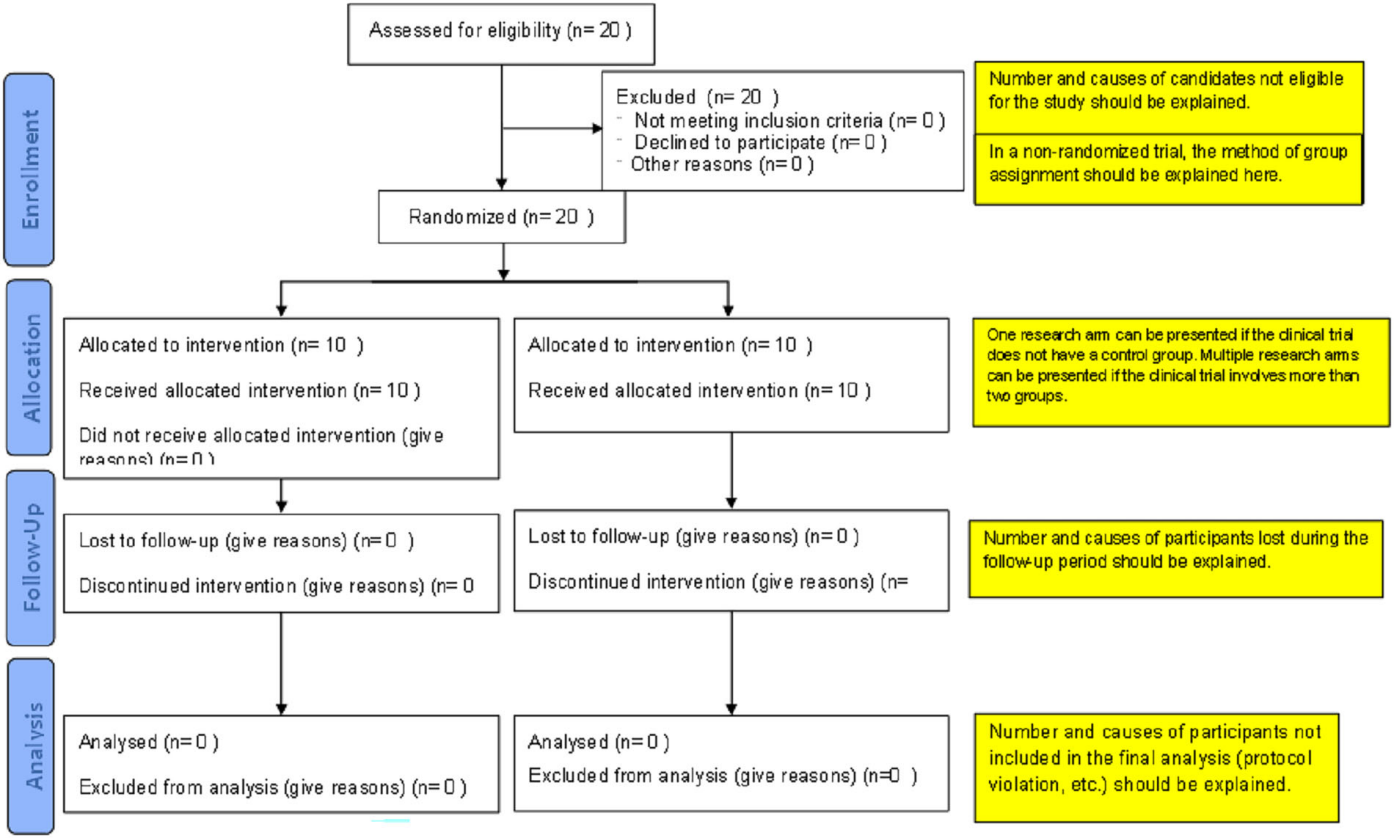
CONSORT 2010 flow diagram (adapted from http://www.consort‐statement.org/).

Retention and transfer tests: In the 17th session, the retention test was administered, and in the 18th session, the transfer test was administered. The retention (Abdollahipour et al. 2017) and transfer tests were exactly like the pretest, with the difference that in the transfer test, the participants were given a lighter ball (Table 4) (Van den Tillaar and Marques 2013). Both quantitative (in terms of points calculated based on the number of bowling pins that fall on the ground (Abdollahipour et al. 2017) and qualitative (the TGMD3 test of the ball throw subscale) tests were performed. The experimental progression from the pretest phase, through training to the post‐test phase, is shown in Figure 1.

**TABLE 4:** Exercises for pretest and post‐test.

	Exercise	Tools	Repetition	Distance of bowling
TGMD3 subscale test of underhand ball throwing	If there is an error in underhand throwing the ball, 0 point will be given to the participant, and if the throw is correct, 1 point will be given to the participant.	10 toy bowling pins Bowling ball	2	3 m
Score	In each throw, points are calculated based on the number of bowling balls that fall to the ground.		2	3 m

*Note*: According to the reference, the subject's distance to the first bowling pin should be 6 m, but because the subjects in this center had not worked on their fundamental motor skills before, they were weak in terms of throwing power, so the distance was gradually reduced until we reached 3 m.

The corresponding author was responsible for enrolling the participants and assigning them to respective interventions.

### 2.4 Statistical Analysis

The descriptive statistics involved the average dispersion indices and standard deviations. The inferential statistics involved paired and independent‐samples *t*‐tests. First, the normality of the data was checked. Then, a series of paired *t*‐tests was run to compare the pretest scores of each group, and independent sample tests were also conducted to compare the post‐test scores of the two groups. By using the Shapiro–Wilk test, the data that were not normal were analyzed by the Wilcoxon test, and the data that were normal were analyzed by the *t*‐test. Also, a significance level of 0.05 was considered in all tests.

## 3 Results

The mean results of qualitative (TGMD3) and quantitative (score) tests are reported in Table 5.

**TABLE 5:** Mean TGMD3 and score tests for both groups in pretest and post‐test.

	Errorless learning group			Analogical group		
	mean ± SD			mean ± SD		
Ulrich Gross Motor Development Test, 3rd Edition (TGMD3).	Pretest	Post‐test		Pretest	Post‐test	
		Retention	Transfer		Retention	Transfer
	1.38 ± 0.744	2.00 ± 1.069	2.03 ± 0.991	1.13 ± 0.641	3.53 ± 0.835	3.41 ± 0.926
Score	Pretest	Post‐test		Pretest	Post‐test	
		Retention	Transfer		Retention	Transfer
	5.18 ± 2.326	14.13 ± 3.834	14.00 ± 3.733	5.25 ± 1.832	13.55 ± 3.742	14.13 ± 3.980

### 3.1 Bowling Skill

Based on paired *t*‐tests, errorless learning and analogical learning with an external focus of attention in retention (*p* = 0.001, *t* = −5.468; *p* = 0.001, *t* = −5.172) and transfer (*p* = 0.001, *t* = −5.480; *p* = 0.001, *t* = −5.940) bowling skills had a significant effect on children with ASD. Also, according to the independent *t*‐test, between errorless learning and analogical learning with an external focus of attention in the retention post‐test (*p* = 0.746, *t* = 0.330) and transfer (*p* = 0.949, *t* = −0.065), there was no significant difference in bowling skills.

### 3.2 Underhand Ball‐Throwing Skills

Based on the Wilcoxon test, errorless learning by an external focus of attention on retention (*p* = 0.414, *z* = −0.816) and transfer (*p* = 0.236, *z* = −1.186) had no significant effect on underhand ball‐throwing skill in children with autism. However, according to the paired *t*‐test, analogical learning by an external focus of attention on retention (*p* = 0.414, *z* = −0.816) and transfer (*p* = 0.236, *z* = −1.186) had a significant effect on the underhand ball throwing skill, and finally, as shown in the Mann–Whitney test, errorless learning and analogical learning by an external focus of attention on retention (*p* = 0.004, *z* = −2.875) and transfer (*p* = 0.030, *z* = −2.165) yielded a significant difference in the underhand ball‐throwing skill in children with autism.

Also, the paired *t*‐test for comparing the pretest and post‐test of the underhand ball‐throwing skill in the analogical learning group showed that there is a significant difference in both retention (*p* = 0.002, *t* = −4.723) and transfer (*p* = 0.001, *t* = −5/293).

The retention post‐test scores of underhand ball throwing in the errorless group failed to characterize a normal distribution, so the Mann–Whitney test was used to compare the post‐test scores of underhand ball throwing in the errorless and analogical learning groups.

Based on the Mann–Whitney test, there is a significant difference in both retention (*p* = 0.004, *z* = −2/875) and transfer (*p* = 0.030, *t* = −2/165).

## 4 Discussion

The present study compared errorless learning and analogical learning approaches with an external focus on bowling skill in children with ASD. Errorless learning can be justified by hypothesis testing. We hypothesized that errorless learning by an external focus of attention had a significant effect on retention and transfer of bowling skills in children with ASD. Hypothesis testing in the early stages of learning involves the identification and correction of errors by skilled learners (Maxwell et al. 2001). By simplifying the learning environment, the errorless learning method reduces the possibility of errors and thus protects the individual from hypothesis testing. Maxwell and Masters (2008), in their study, limited the learning environment to reduce errors, and the results showed that error minimization causes implicit learning that is independent of working memory. In their research, Maxwell et al. (2001) examined the golf swing in errorless and errorful learning environments, and the results indicated that the performance of the participants improved in the errorless group and deteriorated in the errorful group. As reported by the above studies, the errorless learning method showed a positive effect. Errorless practice has been attributed to the acquisition and learning of skills.

The results of the present study also showed that analogical learning by external focus of attention on retention and transferring bowling and underhand ball‐throwing skills has a significant effect. In teaching via the analogical learning approach, it should be noted that the analogy should be in harmony with the culture, understanding, and language of the participants; otherwise, it will lead to improper performance due to a lack of proper understanding (Liao and Masters 2001). The results of the study are in line with the studies conducted by Chatzopoulos et al. (2022) and Lola et al. (2023), which found that the analogical learning group outperformed in the balance task in the study by Chatzopoulos et al. (2022) and resulted in superior accuracy in the study by Lola et al. (2023) (Chatzopoulos et al. 2022; Lola et al. 2023). Also, the results of the present study are consistent with research by Komar et al. (2014), which found that the analogical learning method was used in butterfly swimming training, and the results indicated that this method increased the internal coordination of swimmers and reduced the time of their skill execution. The results also agree with a study by Lola and Tzetzis (2021), which found that the analogical learning group achieved a higher score in the post‐test and retention test than the implicit, explicit, and control groups. Similarly, in a study by Kim, Qu, and Lam (2021), the analogical learning group in the Y balance skill model showed better performance. Also, the findings are in line with the studies by Jeffrey and Lee (2021) and Zeniya and Tanaka (2021), which found that training via the analogical learning method improved the skill score and accuracy (Abdollahipour et al. 2017; Zeniya and Tanaka 2021). In addition, Capio et al. (2020) reported improvements in the skill patterns of the participants. The results were contrary to the results of Poolton et al. (2007) study, wherein the analogy‐inference group had a performance decline in the transfer phase of the dual task. The participants in this study stated that they had not been able to understand the concept of simile properly. Finally, it can be concluded that teaching bowling skills and underhand ball throwing to children can be useful if the similes employed are familiar to the participants and are understood correctly. Based on the results of this research, there was no significant difference between errorless learning and analogical learning approaches with an external focus on the retention and transfer of bowling skills in children with ASD. As a result, the age, position, culture, and status of individuals should be taken into account when using this method. These results were in line with research by North, Warren, and Runswick (2017), which found that the analogical learning and errorless learning (equally) groups showed better performance than the explicit group in golf putt accuracy. Also, these results were contrary to the results reported by Ghaffari's (2014) study, where the effect of analogical learning on retention and transfer of basketball shoot accuracy was significantly higher than that of the errorless group. However, the results of the present study did not show a significant difference. Finally, it can be concluded that the application of errorless learning and analogical learning methods can be effective in teaching bowling skills to children with autism.

Also, in the present study, errorless learning by an external focus of attention did not have a significant effect on retention and transferring the skill of underhand ball throwing. In the present study, in the errorless group, in the pattern (qualitative) section, the examiner and occupational therapist emphasized hitting the target, and as a result, the participants' attention was focused on the target (in the present study, bowling pins), leading to more evident effects in this section (Seyedzadeh 2014). These results are in line with Ghaffari's (2014) research, which indicated that the errorless learning practice had no significant effect on the retention and transfer of the shooting skill pattern of 10‐ to 11‐year‐old girls. On the other hand, the results of the present study are contrary to a research by Capio et al. (2013), which found that errorless training had a significant effect on the skill pattern. It can be concluded that errorless learning does not focus on the movement pattern; therefore, the skill pattern of underhand ball throwing was not affected by errorless training, and the participants did not improve in the transfer and retention tests of the skill pattern.

Also, in the present study, there is a significant difference between errorless learning and analogical learning by external focus of attention when it comes to how well children with ASD have retention and transfer of the underhand ball‐throwing skill. According to the theory of Sparrow and Newell (1998), in errorless practice, increasing accuracy in throwing movements causes automatic control and increases the economy of movements. The results are in line with a study by Tse et al. (2017) wherein the analogical learning group had a better performance in the jump‐rope skill than the explicit group in the retention test. The research by North, Warren, and Runswick (2017) did not agree with the current research results. In their research, they divided the participants into three groups: explicit training, analogical learning, and errorless training, and the results show that the participants in the analogical learning and errorless groups were superior (equally) to the explicit group in the ability to retain performance under the pressure of anxiety and a dual task. In the analogical learning method, it is possible to use the analogy by simplifying the instructions of the skill and placing it in a biomechanical metaphor that includes all the rules and regulations of the execution of the skill, allowing the trainers to learn the best possible way to use it implicitly in skill training. Therefore, in the analogical learning method, the movement pattern is taught as an analogy, along with all the details of the skill, such as the opposite foot of the hand, the continuation of the movement, and so on. It has been taught to the participants in a very detailed way and using analogies. In addition, the impact of errorless training on enhancing accuracy in throwing movements has been analyzed in relation to automatic control, alongside its role in improving movement efficiency and executing actions with reduced energy. Therefore, in the errorless group, the participants were not taught the skill pattern of underhand ball throwing, and more emphasis was placed on omission errors. As a result, it can be expected that there is a difference between the errorless and analogical learning methods, and the analogical learning group will perform better in the underhand ball‐throwing skill pattern than the errorless group.

In both methods of training (errorless learning and analogical learning), the external focus of attention has been used. The advantages of the external focus of attention compared to the internal focus of attention were substantiated in many previous research studies (Abdollahipour et al. 2017; Maxwell and Masters 2002; Wulf 2013; Wulf et al. 2010). Therefore, it can be concluded that the internal focus of attention imposes a greater load on the working memory, and ultimately, it is associated with poorer performance. It can be said that adopting an external focus of attention reduces conscious interference in the processes that control movements and yields better performance and learning (Wulf 2007). These results are in line with the results of the studies conducted by Samsudin and Low (2017) and Asadi et al. (2022). It is important to note that the results of Tse's (2019) study, which was done on children with ASD, go against the results of previous studies and theories that posited the superiority of the internal focus of attention over the external focus of attention. However, in our study on people with ASD, the opposite was true. The results showed that the external focus of attention can be useful for the performance of these children, although few studies have been conducted to address this issue in children with ASD, highlighting the need for further investigations.

The limitations of the present study are acknowledged. For example, it did not consider female participants, had a limited sample size, and failed to examine internal attention as well as the control group. Future studies are recommended to address these issues and use two intervention groups (the analogical learning and errorless groups) along with a control group in order to offer deeper insights into the effects of the focus of attention and implicit training.

## 5 Conclusion

In general, the results of the current research showed that analogical learning with an external focus of attention can be effective in both bowling and underhand ball‐throwing skills in children with ASD. This approach was beneficial when combined with errorless training with an external focus of attention on enhancing bowling skills. However, it was not useful in teaching the underhand ball‐throwing skill to children with ASD. It can be inferred that the analogical learning method employs the most detailed movement patterns, which leads ASD children to learn the underhand ball‐throwing skill more effectively than through the errorless method. This approach enhances their retention, and errorless training tends to highlight specific aspects more prominently. Also, an external focus of attention on both errorless training and analogical learning in children with ASD can lead to better performance. However, more research is needed to arrive at robust evidence.

## Author Contributions

**Mina Khodayari**: writing–original draft, conceptualization, formal analysis, data curation, investigation. **Rasoul Yaali**: conceptualization, methodology, investigation, validation, project administration, supervision, writing–review and editing. **Farhad Ghadiri**: conceptualization, writing–review and editing, investigation.

## Ethics Statement

All procedures performed in this study were in accordance with the ethical standards of the institutional and/or national research committee and with the 1964 Helsinki Declaration and its later amendments or comparable ethical standards.

## Consent

The study was approved by the Institutional Review Board in Kharazmi University. All participants signed an informed consent form.

## Conflicts of Interest

The authors declare no conflicts of interest.

### Peer Review

The peer review history for this article is available at https://publons.com/publon/10.1002/brb3.70139.

## Data Availability

Data are available for scientific use by requesting from the corresponding author.
